# Induction of Low-Level Hydrogen Peroxide Generation by Unbleached Cotton Nonwovens as Potential Wound Dressing Materials

**DOI:** 10.3390/jfb8010009

**Published:** 2017-03-06

**Authors:** J. Vincent Edwards, Nicolette T. Prevost, Sunghyun Nam, Doug Hinchliffe, Brian Condon, Dorne Yager

**Affiliations:** 1 United States Department of Agriculture, Southern Regional Research Center, New Orleans, LA 70124, USA; nicolette.prevost@ars.usda.gov (N.T.P.); Sunghyun.nam@ars.usda.gov (S.N.); doug.hinchliffe@ars.usda.gov (D.H.); brian.condon@ars.usda.gov (B.C.); 2 Plastic and Reconstructive Surgery, Virginia Commonwealth University, Richmond, VA 23111, USA; dorne.yager@vcuhealth.org

**Keywords:** hydrogen peroxide, chronic wounds, cotton, wound dressings, wound healing

## Abstract

Greige cotton is an intact plant fiber. The cuticle and primary cell wall near the outer surface of the cotton fiber contains pectin, peroxidases, superoxide dismutase (SOD), and trace metals, which are associated with hydrogen peroxide (H_2_O_2_) generation during cotton fiber development. Traditionally, the processing of cotton into gauze involves scouring and bleaching processes that remove the components in the cuticle and primary cell wall. The use of unbleached, greige cotton fibers in dressings, has been relatively unexplored. We have recently determined that greige cotton can generate low levels of H_2_O_2_ (5–50 micromolar). Because this may provide advantages for the use of greige cotton-based wound dressings, we have begun to examine this in more detail. Both brown and white cotton varieties were examined in this study. Brown cotton was found to have a relatively higher hydrogen peroxide generation and demonstrated different capacities for H_2_O_2_ generation, varying from 1 to 35 micromolar. The H_2_O_2_ generation capacities of white and brown nonwoven greige cottons were also examined at different process stages with varying chronology and source parameters, from field to nonwoven fiber. The primary cell wall of nonwoven brown cotton appeared very intact, as observed by transmission electron microscopy, and possessed higher pectin levels. The levels of pectin, SOD, and polyphenolics, correlated with H_2_O_2_ generation.

## 1. Introduction

### 1.1. Wound Healing

Wound healing science has made strides toward understanding how wound dressings may be designed to address critical unsolved issues in wound repair and treatment [1]. Two notable milestones in the history of wound dressing development, that have marked a major influence of material design on wound healing, include Lister’s discovery of impregnated antiseptic dressings [2], and one hundred years later, Winter’s landmark paper on moist wound healing [3]. Dressing research and development has been influenced by advances in wound healing science and new concepts of promoting healing [4].

Through the ages, natural wound healing fibers and substances have often been derived from polysaccharides. For example, honey, which is a complex mixture of polysaccharides, was used in ancient cultures, but has recently been developed in chronic wound dressing applications [5]. In addition, oxidized regenerated cellulose, carboxymethylcellulose, and chitosan, *N*-acetyl-glucosamine, are incorporated into materials that constitute structurally- or process-modified polysaccharide fibers used in wound dressings [6,7,8]. However, cotton in its many forms remains the most widely used material that forms the basis of wound dressings. Cotton fibers are complete plant cells that grow from cotton seeds [9]. The fiber is mainly composed of cellulose molecules, which are found in the primary and secondary cell wall, mostly in small crystallites [10]. The crystallites are stabilized by conventional O-H…O hydrogen bonds, as well as the weaker van der Waals forces and C-H…O hydrogen bonds [10]. The morphology of the cotton fiber consists of an outer protective cuticle which contains hydrophobic lipids and pectin, which are associated with the primary cell wall. Both lipids and pectin constitute approximately one-two percent of the weight of the fiber [9]. Conventionally, the components of the cuticle are removed during the chemical processing of cotton. However, when greige cotton is hydroentangled into a nonwoven material the components of the cotton fiber cuticle are retained [11]. 

### 1.2. Greige Cotton

Unbleached cotton (greige cotton), which has also been descriptively identified as fibrous pectic-cellulose, has received increased attention, based on innovations in its processing [12]. Because little water is consumed by this process and it does not involve scouring/bleaching steps, this cotton product is more eco-friendly than conventionally-processed cotton. This mechanical process opens and exposes the hydrophilic cellulosic component of a greige cotton fiber to water absorption [13,14]. The waxy cuticle is loosened or lifted from the fiber, exposing pectin and cellulose associated with the primary cell wall, and rendering the cellulose of the secondary cell wall more accessible. This study was undertaken to determine whether this novel cotton product has properties that would make it amenable to being incorporated into nonwoven absorbent products, such as wound dressings.

The signals generated by reactive oxygen species (ROS) have been shown to play important roles in regulating a number of important processes that occur during repair, including cell migration, proliferation, and differentiation, as well as the synthesis of extracellular matrix components [15]. Low-level hydrogen peroxide generation has been shown to be central to signaling events in the wound healing process [16,17,18]. Wound closure of epithelial tissues and the recovery of blood vessels, are also essential steps in wound healing. ROS and Nicotinamide adenine dinucleotide phosphate (NADPH) oxidases promote the migration and proliferation of cells required for the repair of epithelial tissues and blood vessels [19]. Wound angiogenesis is stimulated by a low concentration of H_2_O_2_ and inhibited by catalase [20]. Low concentrations of H_2_O_2_ at the wound site can rescue repair in NADPH oxidase-deficient mice [21]. Taken together, these results demonstrate that ROS signaling coordinates the function of various cell types during the wound response. 

The vast majority of commercial dressing materials focus on the physical aspects of wounds, e.g., acting as a physical barrier to further injury, providing an optimal level of moisture, and the removal of excess exudate. Dressings with the ability to modulate the wound environment for stimulating healing, would represent a significant advancement in current treatment paradigms. To this end, this study was undertaken to determine whether this novel cotton product might have properties that would make it amenable to its incorporation in nonwoven absorbent products, such as wound dressings. 

## 2. Results

### 2.1. Pectin Levels in Greige Cotton

**Figure 1:**
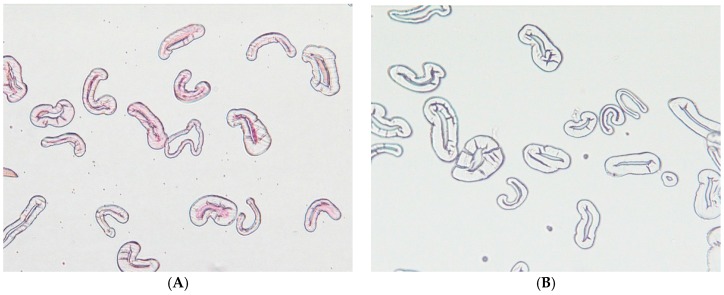
Microscope images of cross-sectioned cotton fibers after staining with Ruthenium Red are (**A**) hydroentangled (HE) brown nonwoven greige cotton and (**B**) scoured and bleached cotton fiber. Note: The brown cotton fiber was distinctively stained.

Ruthenium red staining indicated that greige cotton contains significant levels of pectin, predominately centered over the lumen (Figure 1). As shown in Figure 1, significant staining occurred, even after the process of hydroentanglement, which involves exposure of the cotton fiber to high pressure water jets. 

**Figure 2:**
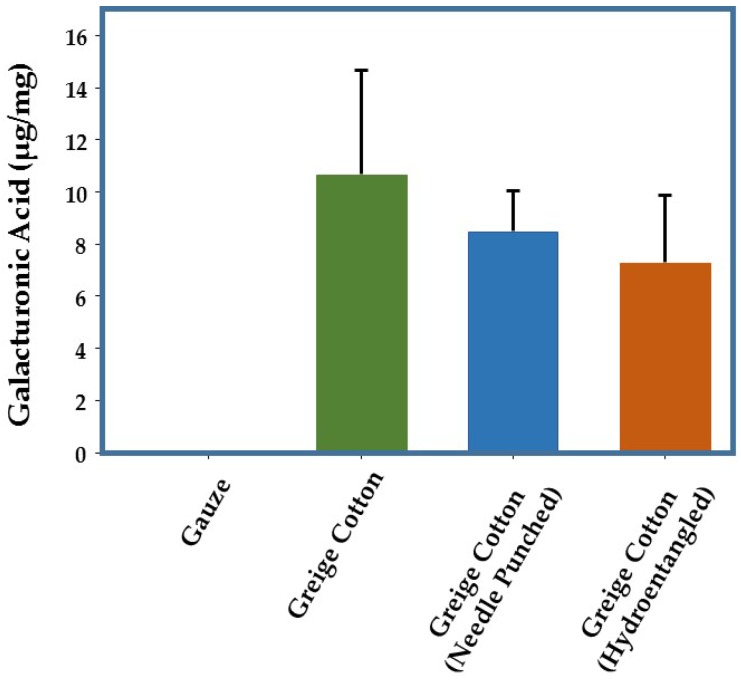
Pectin Levels Associated With Greige Cotton. Galacturonic acid, the primary constituent of pectin, was measured in a number of independent greige cotton samples and compared with the galacturonic acid content in chemically-processed cotton (gauze). Galacturonic acid levels in gauze were undetectable, whereas mean levels in greige cotton were approximately 10 µg/mg of fabric. The effects of processing these cottons into nonwoven materials by needle punching or hydroentanglement, slightly decreased the galacturonic acid content, but this was not statistically significant. Error bars represent one standard deviation.

Pectin is a major plant structural cell wall polysaccharide [22], and has been implicated as a source of plant-based hydrogen peroxide production through both enzymatic and non-enzymatic pathways [22,23,24,25,26]. Pectin demonstrates redox properties through interaction with hydroxyl radicals that yield hydrogen peroxide. In recent years, it has been shown that UV-irradiation of pectin provokes hydroxyl radicals, to produce carbon dioxide radicals from polygalacturonic acid, which in turn, act as a reducing agent to produce superoxide [27]. Superoxide dismutates to hydrogen peroxide, which has been shown to be involved in the induction of primary and secondary cell wall biosynthesis in cotton plants [28,29,30]. Additionally, pectin is a component in a family of dressings associated with low-level hydrogen peroxide generation [31,32]. Because pectins are rich in galacturonic acid, an assay for galacturonic acid was used to provide an indication of pectin levels in various greige cotton samples. As shown in Figure 2 chemically-processed cotton (gauze) possessed no detectable galacturonic acid, whereas seven field cotton samples contained mean levels of 10.7 µg (SD 4.0) of galacturonic acid per milligram of material. Mean levels were slightly lower when processed into nonwoven textiles (hydroentanglement: 7.3 µg/mg SD 2.3; needlepunch: 8.5 µg/mg SD 1.6).

### 2.2. Generation of Hydrogen Peroxide

We next examined the ability of greige cotton derived from numerous strains of *Gossypium hirsutum* to express hydrogen peroxide. These included both naturally brown colored and white varieties. We also examined whether the processing steps for creating nonwoven materials, e.g., needle punching or hydroentangling, might further influence hydrogen peroxide expression.

**Figure 3:**
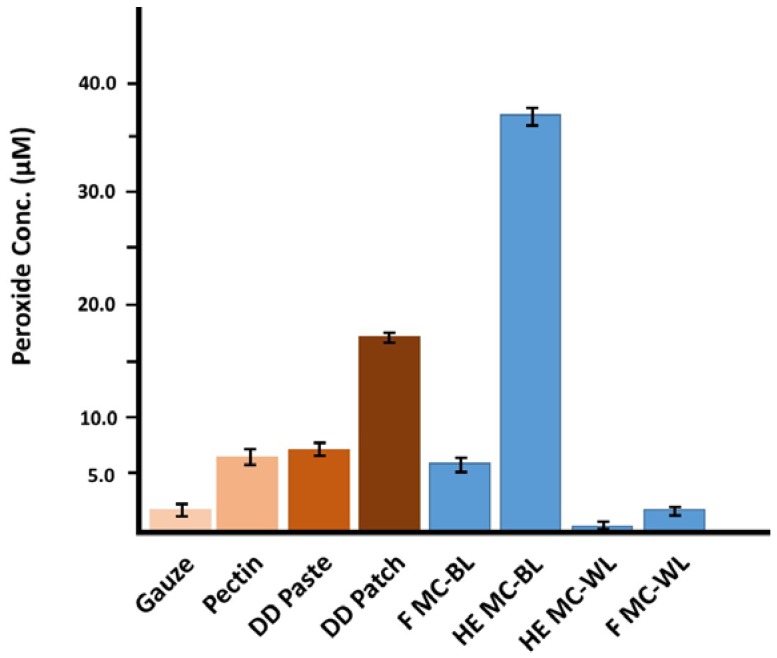
Hydrogen peroxide concentration after ~24 h using the Amplex Red (AR) assay employing horseradish peroxidase (HRP) at final volume concentration of 100 µM of AR and 0.5 U/mL HRP). Hydrogen peroxide concentration after ~24 h (using the Amplex Red (AR) assay at final volume concentration of 100 µM of AR and 0.2 U/mL HRP) of two forms of cotton; field fiber (F) and hydroentangled (HE) ground greige cotton samples (10 mg/mL), pectin and commercial dressings. Commercial dressings included bleached cotton gauze (Gauze), DuoDERM (DD) Paste and DD Patch.

Figure 3 presents examples of levels of hydrogen peroxide generated by 10 mg of finely ground material, incubated overnight in 1 mL H_2_O. Bleached cotton (gauze) was included as a control. Interestingly, while H_2_O_2_ was detected by all of the greige cottons, there were significantly higher levels produced by a brown greige cotton, compared with a white cotton. Processing by needlepunch or hydroentanglement had no significant effect on H_2_O_2_ levels. 

In the presence of catalytic metals, ascorbate can have pro-oxidant effects, where the redox-active metal is reduced by ascorbate, and then in turn, reacts with oxygen, producing superoxide. Superoxide can subsequently spontaneously or enzymatically dismutate, to produce H_2_O_2_. The addition of ascorbate to greige cotton resulted in a significant increase in the production of H_2_O_2_ (Table 1). Ascorbate has also been demonstrated to reduce Cu(II) to Cu(I), and further induce the generation of H_2_O_2_ [33]. However, the addition of Cu(II) sulfate to greige cotton did not induce significant changes in the production of H_2_O_2_. 

**Figure 4:**
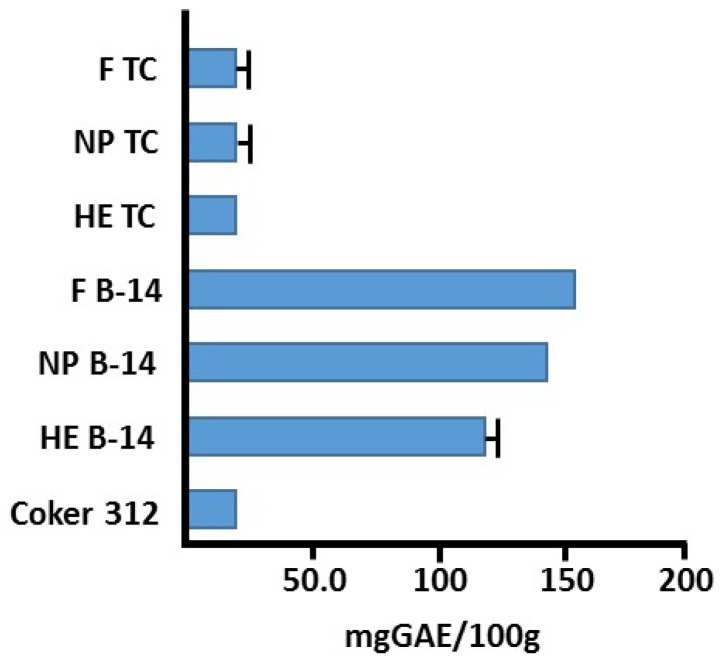
Total phenolic content of brown and white cotton fiber/fabric based on gallic acid equivalents (GAE).

The observed generation of H_2_O_2_ by greige cottons could also be the result of contributions by other factors, such as increased superoxide dismutase activity and/or by an increased presence of polyphenolics [26,29,34]. Interestingly, both white and brown greige cottons exhibited SOD activity, although this was much more pronounced when using the brown cotton (Table 2). Similarly, hydrogen peroxide generation in commercial-based dressings is apparent. Subsequently, it was found that a comparatively high SOD activity (Table 1), as well as a high content of polyphenolics, is present in brown cotton (Figure 4) versus white cotton. Both SOD and polyphenolics are directly linked to the production of hydrogen peroxide. 

### 2.3. Elements Present in Pectin

Element analysis using inductively-coupled plasma mass spectrometry revealed that calcium levels were higher in brown versus white cotton (Table 3). Calcium binding to pectin through coordination with the galacturonic carboxyl induces a conformation change that promotes the adherence of peroxidases to pectin, influencing pectin hydrolysis and the release of ROS [24]. Calcium and other metals including copper, titanium, and iron, have also been shown to mediate ROS and hydrogen peroxide production [35,36,37]. Calcium and hydrogen peroxide are thought to be the earliest signals in both plant and animal healing and defense [17,38]. Calcium also plays a role in cell signal transduction [35,37]. As shown in Table 3, calcium was found to be at higher levels in brown cotton varieties. Higher calcium levels are also consistent with the higher hydrogen peroxide levels observed in the brown cotton samples. However, the higher levels of calcium observed in the hydroentangled cotton, may partially result from calcium in the hydroentanglement process water. It is also worth noting that higher pectin levels presuppose increased amounts of calcium pectate [22,24].

The chemical scission of glycosidic bonds in polygalacturonic acid (the polysaccharide of pectin) by peroxidase-generated hydroxyl radicals, produces hydrogen peroxide in the apoplastic space of plant tissue, as well as the primary cell wall [25]. The plant apoplast, which is a kind of aqueous solution that permeates the living plant, also contains oxygen, ascorbate, and Cu^2+^, which can non-enzymatically produce hydrogen peroxide through trace levels of copper bound to the plant cell wall, combined with equimolar amounts of oxygen and ascorbate [26]. This combination of oxygen, copper, and ascorbate, also readily generates hydroxyl radicals, which in the presence of oxidases, is converted to hydrogen peroxide. As shown in Table 2, copper is present at approximately 2 ppm in most of the field and fabric cotton samples, with the exception of the field and hydroentangled white cotton. Copper in its reduced form (Cu^+^), participates in the Fenton reaction (see below in Equation(1)), where it may react with hydrogen peroxide to form hydroxyl radicals and Cu^2+^. It has been observed that it is 60 times more effective than Fe^2+^ in the Fenton reaction [39]. In light of this, it is noteworthy that iron was found to be present at considerably higher levels than copper in the cotton fibers studied, as shown in Table 2. Thus, it is hypothesized that a cyclical process of hydrogen peroxide and hydroxyl radical generation may enable prolonged coupling of the Fenton reaction free radical hydroxyl product, to the SOD conversion of hydroxyl radicals to hydrogen peroxide.

Cu^+^ + H_2_O_2_ → OH + OH^−^ + Cu^2+^ (1)

### 2.4. Effect of Cotton-Based Hydrogen Peroxide on Dermal Fibroblasts

**Figure 5:**
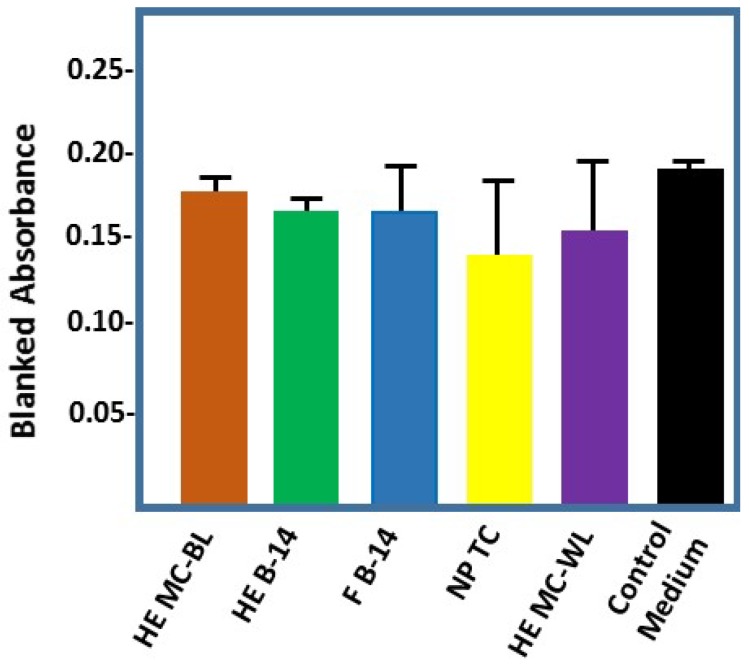
The graph displays the Δ O.D._495nm_ measured after Promega MTS reagent was added to cotton-generated hydrogen peroxide-treated fibroblasts, and incubated for 30 min to assess formazan production. Error bars represent SD.

Despite the destructive potential of ROS, cells have developed defense mechanisms to prevent or limit oxidative injury [17,40]. Preliminary experiments were conducted to measure the effect of cotton-conditioned medium on dermal fibroblasts incubated overnight, seen in Figure 5. Formazan production was measured to assess the hydrogen peroxide cytotoxicity to fibroblasts. Our initial results, as shown in Figure 5, suggest that the hydrogen peroxide produced (levels ranging 5–45 µM in the brown cotton, not shown) is not significantly cytotoxic. This is consistent with Valacchi et al., who have recently demonstrated the non-cytotoxic effects of low-dose ozonated saline on keratinocytes [41]. There were some subtle morphological changes in the fibroblasts after overnight incubations, which may warrant longer incubations.

## 3. Discussion

This paper outlines the low-level hydrogen peroxide generation properties of a number of greige cotton preparations, and examines the relative roles of pectin and primary cell wall enzymes, trace metals, and polyphenolics, in the production of this ROS. In plants, reactive oxygen species (ROS) are well recognized for either being deleterious or beneficial, depending on the concentration and context [40]. In chronic wounds, signals generated by ROS have been shown to play important roles in regulating a number of important processes that occur during repair, including cell migration, proliferation, and differentiation, as well as the synthesis of extracellular matrix components. In this study, we have explored a variety of potential mechanisms for generating hydrogen peroxide in cotton fibers. 

Importantly, we have found that H_2_O_2_ production by greige cottons can be further modulated by the simple addition of ascorbate. The addition of ascorbate to white and brown cotton generated an increase of up to 15 micromolar of hydrogen peroxide. The equimolar combination of ascorbate, copper, and oxygen in the plant apoplast, is known to nonenzymatically produce hydrogen peroxide [26]. Furthermore, it is also notable that ascorbate seems to equalize the hydrogen peroxide activities of white and brown cotton in hydroentangled, needle-punched, and field cotton samples i.e., hydrogen peroxide levels are maintained at a fixed threshold of 12–16 micromolar, that is within the concentration range reported for cell signaling and proliferation.

A consideration of the molecular origins of hydrogen peroxide generation from the cotton is provided by contrasting of two types of cotton fibers (both of Gossypium hirsutum) i.e., naturally colored brown cotton varieties with white cotton varieties. Brown cotton, which has been the subject of specialty utilization, is contrasted with standard white cotton, which has been historically utilized as a scoured and bleached fiber and has been widely used in wound dressing commodities. Recently, some of the beneficial properties of naturally colored brown cotton fibers have been reviewed, and its potential flame-resistant properties have also been suggested [42]. Here, naturally colored brown cotton was found to have a significantly higher capacity for hydrogen peroxide generation at a level that is within the therapeutic window of low level H_2_O_2_ generation, shown to stimulate fibroblast proliferation [43].

Enzymes in plant tissue that play a role in hydrogen peroxide generation and regulation include NADH oxidase, peroxidase, superoxide dismutase, and ascorbate peroxidase. Membrane-bound peroxidases are secretory enzymes that are abundant in the plant cell wall and partly bound to polysaccharides, hence rapid and prolonged hydrogen peroxide release is observed due to membrane-bound peroxidase [35]. In cotton, extracellular Cu/Zn superoxide dismutase (CSD) and peroxidases catalyze the conversion of superoxide to hydrogen peroxide [29,44]. On the other hand, cotton ascorbate peroxidase uses ascorbate as an electron donor to reduce hydrogen peroxide to water, acting as a scavenging enzyme [30].

The hydrogen peroxide generation capacity of both brown and white nonwoven greige cottons may find potential use as a wound dressing application. The potential to stimulate/enhance fibroblast cell proliferation in non-healing wounds, derives from molecular constituents responsible for hydrogen peroxide generation that reside in the cotton fiber primary cell wall, and are not removed as they are with bleached and scoured cotton. 

## 4. Materials and Methods

### 4.1. Materials

The cotton (Gossypium hirsutum L.) fiber (F) samples used were: a brown cotton variety obtained from BC Cotton Inc. (Bakersfield, CA, USA), designated Brown-14 (B-14) harvested ~2005; a mixture of standard upland white cotton varieties from True cotton© (TC©), grown and harvested (2009–2010) in MS; Coker 312 (white cotton variety), grown/harvested 2014; and MC-BL and MC-WL (brown and white of same cotton variety) grown/harvested in 2012 both by Doug Hinchcliffe at SRRC in New Orleans, LA, USA. The nonwoven needle-punched (NP) and hydroentangled (HE) greige cotton sample fabrics used were of the same fibers listed above, and were processed by the SRRC facility. The resulting densities of the hydroentangled fabrics were 120 g/m^2^. The sterile bleached gauze made by MEDLINE was purchased. The scoured and bleached cotton fiber used in microscope imaging was donated by Barnhardt Manufacturing Co., Charlotte, NC, USA. Commercial dressing DuoDerm Signal, and DuoDerm sterile hydroactive paste by Convatec were purchased. Pectin from citrus peel, poly-galacturonic acid (PGA), horseradish peroxidase type I, catalase from bovine liver, d-(+)-galacturonic acid monohydrate, Viscozyme L, gallic acid, Fast Blue RR salt (FBRR) [4-benzyolamino-2,5-dimethoxybenzenediazonium chloride hemi(zinc chloride salt], superoxide dismutase from bovine erythrocyte (SOD), and SOD determination kit (cat #19160) were purchased from Sigma Aldrich (St. Louis, MO, USA). Amplex Red reagent and Amplex Red^®^ Hydrogen Peroxide/Peroxidase Assay Kit cat # A22188 were purchased from Life Technologies (Invitrogen/Molecular Probes, Eugene, OR, USA). Micro bio-spin columns and Econo-Pac 10DG desalting columns containing Biogel P-6DG were purchased from Bio-Rad (Hercules, CA, USA). A novel tetrazolium compound, 3-(4,5-dimethylthiazol-2-yl)-5-(3-carboxymethoxyphenyl)-2-(4-sulfophenyl)-2H-tetrazolium, and inner salt (MTS), were purchased from Promega (Madison, WI, USA). All other chemicals were of a commercial reagent grade and used without further purification. 

### 4.2. Methods

The greige cotton fiber (F) and fabric samples, needled-punched (NP) and hydroentangled (HE), were ground with a Wiley Mill to a 40–80 mesh powder and stored in a container until use. The dry ground samples were stored at room temperature.

### 4.3. Enzymatic Pectin Hydrolysis

Viscozyme L was desalted twice with Bio-Rad Econo-Pac 10DG desalting columns containing Biogel P6DG, using the general protocol listed in the accompanied instruction manual. Desalted viscozyme was added to the reaction volume, to equal a final concentration of 1% solution. For heterogeneous samples, solid fabric of 100 mg was placed in 10 mL 1% viscozyme solution, made with 25 mM sodium acetate at pH 4.8. For homogenous solutions such as pectin and DuoDerm paste, 0.16% (*w*/*v*) solution was made in 25 mM sodium acetate at pH 4.8, and 9.9 mL and 0.1 mL of desalted viscozyme L was added. To all solutions, ~10 mg of ethylenediaminetetraacetic acid (EDTA) was added. The samples were sonicated for 30 min and heated overnight (for 24 h), in a 40 °C water bath. Note: To stop the enzyme action for measuring specific lengths of time, the sample solutions can be heated at 100 °C for 4 min.

### 4.4. Galacturonic Acid Assay

A procedure with minor changes, as described previously, was followed [45]. Briefly, a buffered copper solution was prepared by adding 23.2 g of NaCl, 3.2 g sodium acetate, and 1.0 mL glacial acetic acid to 80 mL of water, stirring to dissolve, before adding 0.5 g copper sulfate, adjusting the pH to 4.8 with NaOH. The final volume was then brought to 100 mL by adding deionized water. For the assay, a standard curve was made of galacturonic acid, with a concentration ranging from 0 to 200 µg/mL. Equal volumes of the various standard concentrations and the buffered copper solution, and the sample solution and buffered copper solution, of 100 µL, were placed in a test tube with glass beads on top, and heated in a 100 °C dry bath for 30 min before removing. A total of 0.8 mL of the diluted Folin-Ciocalteau reagent was added, prepared by mixing 1 mL of 2N Folin-Ciocalteau reagent with 39 mL of water. Color immediately forms; the standards and samples are transferred to a microtiter plate and the absorbance is measured with a microplate reader at 750 nm.

### 4.5. Hydrogen Peroxide Determination Using Amplex Red Assay

For sample preparation, a ground sample was weighed and placed in a microcentrifuge tube with 1 mL of 0.05 M sodium phosphate buffer solution, pH 7.4, giving a concentration of 50 mg/mL of stock solution. This stock solution was vortexed and then sonicated for 10–20 min in an iced water bath. They were centrifuged at 10 K rpm for 30 s to a minute, and the supernatant was used to make a sample concentration of 10 mg/mL, diluted with phosphate buffer.

Working solutions of Amplex Red reagent (AR) of 400 µM, and horseradish peroxidase (HRP) (2 U/mL) placed in assay buffer (0.05 M sodium phosphate pH 7.4), were prepared from an aliquot of AR (20 mM) in dimethyl sulfoxide (DMSO) and a stock solution of HRP 50U/mL, respectively. The standard curve was prepared using 3% H_2_O_2_ and diluted to a working concentration in the range of 40–0 µM. The final concentration is two-fold lower. The hydrogen peroxide standard, sample, and controls, were added to their respective microplate well in duplicate, at 100 µL. To this, 50 µL of AR working solution was added to each, in dim room light. To start the reaction, 50 µL of HRP was added to each well, producing a final assay volume totaling 200 µL, noting that the AR/HRP concentration will be four-fold lower. The reaction was monitored by fluorescence measurement using the microplate reader Synergy-HT exciting at 530 nm/30 and detecting emission at 590 nm/30. The reaction is continuous; measurements were taken at a varied time length. 

### 4.6. Superoxide Dismutase (SOD) Activity

The superoxide dismutase activity was determined using a SOD determination kit Sigma Aldrich cat. #19160. Also known as a SOD Assay Kit-WST, it utilizes Dojindo’s highly water-soluble tetrazolium salt (WST), that produces a water-soluble formazan dye upon reduction with a superoxide anion. The rate is linearly related to xanthine oxidase (XO) activity. The protocol included in the kit was followed with minor changes to sample preparations. Briefly, 50 milligrams of ground sample was placed in a microcentrifuge tube with 1 mL of chilled 0.05 M sodium phosphate buffer, pH 7.4. It was vortexed for mixing and then placed in a sonicator filled with iced water (~5 °C) for 10 min. Following this, samples were centrifuged for 15 min at a low speed. Working solutions of the WST formazan, 2-(4-Iodophenyl)-3-(4-nitrophenyl)-5-(2,4-disulfophenyl) -2H-tetrazolium, monosodium salt, xanthine oxidase, were prepared per instructions. SOD standard solution was also prepared to monitor the assay (not included in the kit). The SOD stock solution of 1000 U/mL was prepared from aliquot in dilution buffer included in the kit, and was further diluted with dilution buffer to prepare a 200–0.001 U/mL range. To start the assay in triplicate, 20 µL of sample or SOD solution was added to its respective well and the sample was added to the blank 2 well. A separate blank 2 well was made for each cotton sample that contained a visible color before the start of the reaction, as with the brown cotton samples. Twenty microliters of Millipore water were added to blank well 1 and 3. The working WST solution, 200 µL, was added to each well and mixed. A dilution buffer of 20 µL was added to the blank 2 and blank 3 well. To start the reaction, 20 µL of the enzyme working solution was added to each sample, the SOD dilution, and blank 1 well, and was mixed thoroughly. The microplate was incubated for 20 min at 37 °C. The absorbance was measured at 450 nm using a microplate reader. The SOD activity (inhibition rate%) was calculated using the following Equation:

SOD activity (inhibition rate%) = [[(A_blank 1_ − A_blank 3_) − (A_sample_ − A_blank 2_)]/(A_blank 1_ − A_blank 3_)] × 100 (2)

### 4.7. Total Phenolic Content via Fast Blue RR (FBRR) Method

The total phenolic content was determined using a modified procedure of Medina and Lester [46,47]. A total of 1 mL of 0.05 M sodium phosphate buffer (pH 7.4) was added to a microcentrifuge tube filled with 50 mg of ground cotton fibers, ~80 mesh. The tubes of samples were vortexed, sonicated in an ice bath for an hour, and then centrifuged for at least 5 min. The supernatant was used in the assay. Gallic acid calibration standards of concentrations ranging from 25–500 µg/mL were prepared. One milliliter of the diluted sample supernatant (1:9) with millipore water, gallic acid (1:9), or millipore water used for blank, was added to a small test tube, followed by 0.1 mL of 0.1% FBRR (4-benzyolamino-2,5-dimethoxybenzenediazonium chloride hemi(zinc chloride) salt), and was then mixed. After a minute, 0.1 mL of 5% sodium hydroxide (NaOH) was added to each tube (sample and standard), mixed, and the reaction was allowed to complete at room temperature, for 60–90 min. Aliquots (200 µL) of each sample/standard were transferred to a microtiter plate in duplicate and the absorbance was measured at 420 nm. A calculation of the total phenolic content was derived from the gallic acid standards measured in µg/mL, and the results of the samples tested were expressed as gallic equivalents, mg GAE/100 g of sample.

### 4.8. Optical Microscope: Sample Preparation and Imaging

A cross-section of the fiber was observed using the techniques developed at the Southern Regional Research Center, which are described in the literature [48,49]. Briefly, a bundle of combed fibers was immersed in a methacrylate matrix solution, and the matrix was polymerized in a Teflon tubing under UV light for 30 min. After removing the block of fiber bundle from the tubing, the block was re-embedded in BEEM^®^ (Ted Pella, Redding, CA, USA) embedding electron microscope capsules. The sample was sliced into thin sections (1 µm) using a PowerTome Ultramicrotome (Boeckeler Instruments, Inc., Tucson, AZ, USA). These sections were placed on a glass slide, and the embedding medium was removed using methyl ethyl ketone in a glass Petri dish. The sample was then stained with an aqueous solution of Ruthenium red (0.02 wt %) for 30 min, and washed with deionized water multiple times. The image was taken with an optical microscope (Olympus BX WI, Waltham, MA, USA). 

### 4.9. ICP/MS

Cotton fiber/fabric samples, of 0.5 g, were first digested in acid, 10 mL of 50% nitric acid, using a CEM Corporation MARS 6 Microwave Reaction System (Matthews, NC, USA), employing a standard digestion method. Trace metal analysis was performed by The Coordinated Instrumentation Facility (CIF) of Tulane University in New Orleans, LA, USA, using a ThermoFisher Element2™ high-resolution inductively-coupled plasma mass spectrometer (ICP-MS). Two sample dilutions were performed. The initial stock sample solution, of 10 mL, was diluted to 50 mL using DI water. To 100 µL of the sample, 19.9 mL of 2% nitric acid, 20 µL of internal standard, and 1 ppm of Scandium were added, for a dilution factor of 200. Samples were introduced using an ESI FAST auto sampler with a 1 mL loop and Scott spray chamber. The eluent was 2% nitric acid. The take-up time was 1.5 min and wash time was 10 s. All elements were analyzed at a medium mass resolution (>4000), to eliminate potential interferences from polyatomic species. The mass spectrometer performed three runs of five passes and reported each average value with an instrument relative standard deviation (RSD). The calibration range for Ca, Mg, and Fe was 0.78125–625 ppb, for Cu was 1.40625–1125 ppb, and was 1.015625–812.5 ppb for Mn and Zn. 

### 4.10. Fibroblast Cytotoxicity Assay

To the ground cotton fiber/fabric samples, of 50 mg, a total of 1 mL of Dulbecco’s Modified Eagle’s Medium (DMEM) without bovine serum, was added. Initially, 0.5 mL of medium was added to the samples and allowed to sit for a few min after vortexing. An additional 0.5 mL of medium was then added, the mixtures were vortexed for 2 min, and were finally sonicated for 10 min. Then, the samples were incubated in the dark overnight. Samples were centrifuged at ~20,000× *g* at 4 °C for 15 min. Serum was added to ~500 µL of the samples’ supernatant, equaling a final concentration of 2%. In triplicate, 100 µL of the serum containing supernatant was added to confluent fibroblasts in a 96-well microplate. The plates were incubated overnight in a carbon dioxide (CO_2_) incubator. Next, Promega MTS reagent, of 5 µL, was added to the treated fibroblasts. An initial optical density (O.D.) was measured at 495 nm, the plate was incubated for 30 min, and the O.D. was measured again at 495 nm. To calculate the formazan produced, the initial O.D. for each well was subtracted from its final O.D. 

## Figures and Tables

**Table 1:** Influence of Cu(II) or Ascorbate on Hydrogen Peroxide Generation (µM).

Sample	Alone	+0.2 mM Cu (II)	+0.2 mM Ascorbate
F-TC (white)	1.0	0.4	15.5
NP-TC	0.8	0.4	14.5
HE-TC	0.6	ND	12.5
F-B14 (brown)	2.5	3.0	17.2
NP-B14	2.2	2.8	17.0
HE-B14	2.8	3.0	15.2
F-Coker312 (white)	0.8	1.2	17.4

F—Field fiber; NP—needlepunched; HE—hydroentangled. ND: not detected. (Alone): Hydrogen peroxide concentration after ~24 h (using the Amplex Red (AR) assay employing horseradish peroxidase (HRP) at final volume concentration of 100 µM of AR and 0.5 U/mL or 0.2 U/mL HRP) of all three forms of cotton, fiber (F) needled punched (NP), and hydroentangled (HE) ground greige cotton samples (10 mg/mL). (Copper and Ascorbate additions): The ascorbic acid- and CuSO_4_ –peroxide-generated values are the result of treated sample minus ascorbic and copper(II) sulfate controls, respectively, (interpolated from standard curve).

**Table 2:** Superoxide Dismutase Assay.

Sample Name	Inhibition Rate%	SOD Activity (U/mL)
F TC (White)	12.43	0.09
NP TC©	10.36	0.08
HE TC©	3.45	0.05
F B-14 (Brown)	63.15	1.86
NP B-14	72.79	3.20
HE B-14	61.32	1.64
F Coker 312 (White)	7.68	0.07

**Table 3:** ICP-MS Data * of Brown and White Cotton.

Sample	^24^Mg	^44^Ca	^55^Mn	^56^Fe	^63^Cu	^66^Zn
F Coker 312	1281.4	347.7	3.7	8.7	0.8	2.3
F TC	544.2	411.0	3.7	379.9	2.1	5.4
F B-14	614.8	601.1	7.3	360.4	2.4	59.7
NP TC©	670.5	542.5	4.5	24.1	1.9	7.0
NP B-14	1069.7	955.0	5.1	23.9	2.2	8.7
HE TC	297.0	857.4	7.4	14.5	0.8	3.5
HE B-14	846.8	1457.3	5.0	18.1	1.8	7.2

* Sample solution mean (ppm) and standard deviation ranged from 0.4% to 5%.
